# Knowledge, Attitude, and Practice of Infection Control and Waste Management among Traditional Medicine Practitioners in Bhutan, 2019: A Nationwide Cross-Sectional Survey

**DOI:** 10.1155/2021/6691780

**Published:** 2021-04-12

**Authors:** Dorji Gyeltshen, Thinley Dorji, Sonam Choda, Chencho Gyeltshen, Sangay Dorji, Tashi Dorji, Ugyen Tshering, Diki Wangmo, Krit Pongpirul

**Affiliations:** ^1^Department of Traditional Medicine Services, Ministry of Health, Thimphu, Bhutan; ^2^Kidu Mobile Medical Unit, His Majesty's People's Project, Thimphu, Bhutan; ^3^National Traditional Medicine Hospital, Ministry of Health, Thimphu, Bhutan; ^4^Khesar Gyalpo University of Medical Sciences of Bhutan, Thimphu, Bhutan; ^5^Department of Preventive and Social Medicine, Faculty of Medicine, Chulalongkorn University, Bangkok, Thailand

## Abstract

**Background:**

In Bhutan, Traditional Medicine (TM) is a part of the government-sponsored free healthcare system and Traditional Medicine Units (TMUs) are colocated with allopathic hospitals. Prevention of healthcare-associated infections and patient safety must apply to all institutionalized healthcare settings including TMUs. This study assessed the knowledge, attitude, and practice of TM practitioners in Bhutan in the field of infection control and waste management practices.

**Methods:**

This was a descriptive study among TM practitioners selected through simple random sampling. Data were collected using a structured pro forma and entered in EpiData 3.1 and analyzed in STATA 13.1.

**Results:**

There were 132 respondents (response rate 98%). The majority (64%) knew the seven steps of handwashing but their knowledge of WHO's Five Moments for Handwashing was poor, especially handwashing after aseptic procedures (17%) and handwashing after touching patient surroundings (5%). Handwashing before palpation of the pulse (37%) and using gloves while dispensing medicines (9%) were poor; but the proportions of handwashing before performing moxibustion (96%), correct disposal of sharps (84%), and disinfection of cupping sets (78%) were high. The majority of participants hold a positive attitude towards the adoption of infection control and waste management practices for the benefit of patients. Only 23% had received preservice and 44% had received in-service training on infection control.

**Conclusions:**

The knowledge and practices of infection control and waste management are optimal only in select domains of practice. The practitioners hold a positive attitude towards the adoption of infection control and waste management standards.

## 1. Background

Complementary and alternative medicine (CAM) continues to offer therapeutic services and is sought after by an increasing number of persons for various diseases and conditions [1]. While CAM is often not covered by insurance systems in the west [1], in Bhutan, a small Himalayan country in South Asia, the Bhutanese Traditional Medicine (BTM; *Sowa Rigpa*), a form of alternative medicine, remains popular and is integrated into its free healthcare system [2, 3]. The Traditional Medicine Units (TMUs) are colocated with allopathic hospitals and offer their age-old treatment recipes and healing procedures [4, 5]. BTM drug recipe not only follows its traditional methods of identification, collection, and manufacturing of herbal plants but also follows modern methods of quality assurance in drug manufacturing, storage, and distribution [6]. Surgical therapies include bloodletting, oil and heat moxibustion, cupping, and the indigenous gold needle therapy that generate sharps and infectious wastes.

The prevention and control of hospital-acquired infections and the appropriate management of infectious wastes are important in institutionalized TM services such as that in Bhutan. The prevalence of healthcare-associated infection in developing countries is 15.5 per 100 patients [7] with a potential for a 35–55% reduction in the rates if multifaceted infection prevention and control measures are implemented [8]. While the burden of hospital-acquired infections in the institutionalized TM system is unknown, the Bhutanese government has instituted good infection control and waste management practices in healthcare settings through the National Infection Control and Health Care Waste Care Management Program since 2004 [9]. The Department of Traditional Medicine Services (DTMS) endorsed this policy through the Standard Operating Procedures adopted in 2012 for all BTM practitioners [10]. This study was conducted to assess the knowledge, attitude, and practices of infection control and waste management measures amongst the Traditional Medicine practitioners of Bhutan in 2019.

## 2. Methods

### 2.1. Study Design

This was a cross-sectional study conducted among TM practitioners in Bhutan and is reported using the STROBE guidelines [11, 12].

### 2.2. Setting

In Bhutan, the healthcare system is built on three-tiered hospital levels: basic health units at the primary level, general and district hospitals at the secondary level, and the referral and the National Referral Hospitals at the tertiary level [2]. TMUs are colocated with allopathic hospitals at all levels except the National Traditional Medicine Hospital—the largest BTM teaching hospital. As of 2019, there were 66 TMUs with 53 *drungtsho* and 113 *menpa* in the country [4].

The health human resources for the BTM system are trained within the country at the Faculty of Traditional Medicine, Khesar Gyalpo University of Medical Sciences of Bhutan, the only medical university in the country [5]. The Faculty of Traditional Medicine provides a three-year diploma course in BTM (*menpa*) and a five-year bachelor course (*drungtsho*). Since 2019, the faculty had instituted MD in several fields of BTM. The graduates are licensed for practice by the Bhutan Medical and Health Council under the BTM category. Some graduates practice in wellness and healing services in the tourism and hospitality sector and outside the government healthcare system.

### 2.3. Study Period

Data were collected between 01 January and 30 June 2019.

### 2.4. Study Population

All TM practitioners were eligible to participate in the study.

### 2.5. Sample Size

In the absence of any sort of assessment in this field, we assumed that 50% of the respondents would have good knowledge of infection control and waste management. The sample size was calculated for proportions considering 95% confidence level, 5% margin of error, 15% dropout rate, and finite correction for 166; the final sample size was 135.

### 2.6. Sampling and Data Collection Method

A simple random sampling method was used to select the required number of participants using a random sequence generator using Microsoft Excel. The study questionnaire and the consent forms were delivered by the DTMS via post/e-mail to those selected practitioners. The filled questionnaires were returned to the principal investigator at a later date.

### 2.7. Study Tool

The data was collected using a self-administered questionnaire in Dzongkha—Bhutan's national language. It contained four sections: sociodemographic and professional details, knowledge, attitudes, and practices on infection control and waste management within the context of the practice of BTM. The study tool was designed and moderated amongst *drungtsho*, *menpa*, and research experts from within the country at an operational research course in 2018 [13]. The study questionnaire was initially drafted in English (see supplementary file) and forward and backward translations were done involving a translation committee at the protocol development workshop. The study tool was pretested among 10 practitioners who were subsequently excluded from the random sequence generated for sampling.

### 2.8. Data Management

The questionnaire was coded and double entered into EpiData Entry version 3.1 (EpiData Association, Odense, Denmark) and analyzed in STATA version 13.1 (StataCorp, 2013; Stata Statistical Software: Release 13; College Station, TX : StataCorp LP). Descriptive statistics are used to present the study findings.

## 3. Results

There were 132 valid questionnaires returned (response rate of 98%). The mean age of participants was 32 (±7) years and the mean duration of work experience was 6 (±5) years; 81 (62%) participants were males. There were 30 (23%) participants who had received preservice training and 58 (44%) had received in-service training on infection control and waste management. The details of the basic characters of the study participants are shown in Table 1.

There were 83 (64%) participants who knew the seven steps of handwashing. As shown in Table 2, the knowledge on the WHO's five moments of handwashing was poor: only 23 (17%) participants knew that handwashing is important before performing aseptic procedures and only six (5%) knew that handwashing should be done after touching patient surroundings. The details of knowledge on infection control and waste management are shown in Table 2.

The majority of participants (124, 95%) held a positive attitude towards learning about infection control and waste management in line with the six ethical principles for BTM practitioners. There were 117 (90%) participants who were interested in adopting all principles of infection control and waste management in their TMUs. The details of the assessment on attitude rating statements are shown in Table 3.

Although the use of the white apron was common (107, 82%), the practice of handwashing was reported only by 48 (37%) during the palpation of pulse and by 85 (66%) during performing moxibustion. The correct disposal of sharps generated from procedures was reported by 70 (84%) and the disinfection of cupping sets was reported by 77 (78%). The practice of disinfecting the TMU premises was reported only by 37 (29%) participants. The details of the practices are shown in Table 4.

## 4. Discussion

This is the first assessment of the knowledge, attitude, and practice of infection control and waste management system in institutionalized TM services in Bhutan. The knowledge of infection control and waste management was good in the majority of the participants and reflects the adoption of allopathic standards within the Traditional Medicine system. The majority knew the seven steps of handwashing promoted by the World Health Organization. However, the participants were not familiar with some of the key moments in handwashing, for example, after performing aseptic procedures and after touching a patient's surroundings. The practitioners may not have been familiar with the latter two because there were no in-patient settings within the BTM settings until 2018.

In our study, only 23% had received training on infection control and waste management as a part of their undergraduate training as this topic was incorporated into the curriculum only in the recent past. Among the senior practitioners, only 44% had received in-service training reflecting the need for additional efforts required in training them. Trainings on infection control are shown to increase the healthcare practitioner's knowledge compared to no interventions [14]. In our study, the participant's knowledge of the spread of hospital-acquired microorganisms was poor as the microbiology curriculum is not a part of their training. Training must focus on key practice points such as the disposal of sharps and biomedical wastes according to standard guidelines.

The practitioners are guided by the six ethical principles in BTM and they hold a strongly positive attitude to adopt infection control and waste management practices. This is receptivity for allopathic principles of infection control among the TM practitioners are an important avenue for intervention. Higher knowledge of infection control is linked with positive attitudes and higher compliance in practice [14, 15]. As a part of the government's effort to prevent the spread of COVID-19 in Bhutan in 2020, all hospitals including the TMUs adopted infection prevention and control measures, and all health workers including TM practitioners were provided with training. This study conducted in 2019 has led to a change in attitudes and practices among TM practitioners and is expected to reduce healthcare-associated infections related to the TM practices.

The prevalence of healthcare-associated infections is high in developing countries and involves the urinary tract, surgical wounds, respiratory system, and bloodstream [7]. In a survey at the National Referral Hospital in Bhutan, the proportion of surgical site infections reported after surgery was high (30.7%) compared to global and regional standards [16]. Another qualitative study reported postsurgery wound infections and urinary tract infections (due to nonadherence to sterile technique during catheterization) as the two main healthcare-associated infections in Bhutan [17]. BTM offers invasive surgical therapies that require compliance with universal precautions. Infection prevention policies and patient safety measures must apply to all forms of institutionalized health facilities in the country [18].

## 5. Limitations

The assessment of attitude and practices could have been influenced by social desirability bias; future studies with on-site audits of practices are required. A qualitative study on the contextualization of allopathic standards in TM practices may provide a real-world scenario on the attitude and practices on infection control and waste management. This study did not assess the knowledge, attitude, and practices of those TM practitioners working in the tourism and hospitality sector and those practiced outside the government sector.

## 6. Conclusions

The knowledge and practices of infection control and waste management are optimal only in select domains of practice. The practitioners hold a positive attitude towards the adoption of national infection control and waste management standards.

## Figures and Tables

**Table 1:** Basic sociodemographic characters of the Bhutanese Traditional Medicine practitioners interviewed for the KAP survey on infection control and waste management, 2019 (*n* = 132).

Basic characters	*n*	(%)
Age group^1^		
18–24 years	7	(5)
25–34 years	93	(72)
35–44 years	21	(16)
45–54 years	8	(6)
55–64 years	1	(0)

Sex^2^		
Male	81	(62)
Female	50	(38)

Work experience		
<5 years	62	(47)
5–9 years	34	(26)
10–19 years	27	(20)
20–29 years	5	(4)
≥30 years	4	(3)

Level of hospital^2^		
National Traditional Medicine Hospital	23	(18)
Regional Referral Hospital	7	(5)
Dzongkhag Hospital	47	(36)
Basic Health Unit	54	(41)

Handwashing facility at the Traditional Medicine Unit*∗*		
Running water	110	(84)
Soap	104	(80)
Sink/washbasin	91	(72)

Preservice training on infection control	30	(23)
In-service training on infection control	58	(44)

^1^Missing = 2. ^2^Missing = 1.

*∗*Multiple responses allowed. The superscripts represent the number of missing data for each variable.

**Table 2:** Knowledge on infection control and waste management among Bhutanese Traditional Medicine practitioners interviewed for the KAP survey on infection control and waste management, 2019 (*n* = 132).

Knowledge parameters	Responses	*n*	(%)
How many steps are there in handwashing in the World Health Organization guideline?^1^	Correct	83	(64)
	Incorrect	46	(36)

Five moments of handwashing			
Before touching a patient	Yes	70	(53)
	No	62	(47)
Before clean/aseptic procedures	Yes	23	(17)
	No	109	(83)
After body fluid exposure/risk	Yes	63	(48)
	No	69	(52)
After touching a patient	Yes	96	(73)
	No	36	(27)
After touching patient surroundings.	Yes	6	(5)
	No	126	(95)

If you are performing a bloodletting procedure, what type of gloves will you use in an ideal situation?^2^	Correct	62	(73)
	Incorrect	23	(27)
After performing a bloodletting procedure, where will you dispose your scalpel?^3^	Correct	10	(12)
	Incorrect	74	(88)
After performing a bloodletting procedure, where will you dispose the gauze material?^3^	Correct	65	(78)
	Incorrect	19	(22)
After performing a bloodletting procedure, what is the first step before you dispose the blood?^4^	Correct	66	(80)
	Incorrect	16	(20)

Which of the following steps in traditional medicine can spread microorganisms?^5^			
Measuring pulse	Yes	10	(8)
	No	111	(92)
Bloodletting	Yes	110	(91)
	No	11	(9)
Dispensing medicine	Yes	2	(2)
	No	119	(98)
Cupping	Yes	36	(30)
	No	85	(70)
Gold needle therapy	Yes	24	(20)
	No	97	(80)

Which of the following are NOT personal protective equipment?^6^			
Mask	Yes	6	(5)
	No	124	(95)
Patient registers	Yes	95	(73)
	No	34	(27)
Medicines	Yes	41	(32)
	No	89	(68)
Gloves	Yes	7	(5)
	No	123	(95)

^1^Missing = 3. ^2^Missing = 47. ^3^Missing = 48. ^4^Missing = 48. ^4^Missing = 50. ^5^Missing = 11. ^6^Missing = 2.

The superscripts represent the number of missing data for each variable.

**Table 3:** Attitude ratings on infection control among Bhutanese Traditional Medicine practitioners interviewed for the KAP survey on infection control and waste management, 2019 (*n* = 132).

Attitude parameters	Not interested		Not sure		Very much interested	
	*n*	(%)	*n*	(%)	*n*	(%)
According to six ethical principles in Bhutanese Traditional Medicine, how interested are you in learning about infection control?^1^	3	(2)	4	(3)	124	(95)
Are you interested in adopting the national guideline on infection control at your Traditional Medicine Unit?^2^	4	(3)	9	(7)	117	(90)
If there is a training on infection control, are you interested to participate in it?^3^	4	(3)	1	(1)	126	(96)

^1^Missing = 1. ^2^Missing = 2, ^3^Missing = 1. The superscripts represent the number of missing data for each variable.

**Table 4:** Practice on infection control among Bhutanese traditional medicine practitioners interviewed for the KAP survey on Infection control and waste management, 2019 (*n* = 132).

Practice parameters	Never		Sometimes		Always	
	*n*	(%)	*n*	(%)	*n*	(%)
How often do you wear apron at your workplace?^1^	1	(1)	23	(18)	107	(82)
Before you read the pulse of a patient, how often do you wash your hands?^2^	22	(17)	60	(46)	48	(37)
Before performing therapies, how often do you wash your hands?^3^	15	(12)	29	(22)	85	(66)
How often do you wash hands after performing moxibustion?^4^	2	(2)	3	(2)	122	(96)
How often do you dispose the scalpels in the correct dustbin?^5^	12	(14)	1	(1)	70	(84)
Do you use a mask when you are suffering from common cold?^2^	3	(2)	41	(32)	86	(66)
Do you use gloves when you dispense medicines?^2^	60	(46)	52	(40)	18	(14)
Do you disinfect the instrument set used for cupping?^6^	3	(3)	19	(19)	77	(78)
Do you examine patients in places other than your chamber?^3^	75	(58)	43	(33)	11	(9)
Do you disinfect the floor of your Traditional Medicine Unit?^3^	22	(17)	70	(54)	37	(29)

^1^Missing = 1. ^2^Missing = 2. ^3^Missing = 3. ^4^Missing = 5. ^5^Missing = 49. ^6^Missing = 33. The superscripts represent the number of missing data for each variable.

## Data Availability

The datasets generated and/or analyzed during the current study are available from the corresponding author upon request.

## Abbreviations

BTM: Bhutanese Traditional Medicine
CAM: Complementary and alternative medicine
DTMS: Department of Traditional Medicine Services
TM: Traditional Medicine
TMUs: Traditional Medicine Units.

## Supplementary Materials

Supplementary Materials Study questionnaire of BTM infection control and waste management survey, 2019.
