# Supplementary material for: Knowledge, Attitude, and Practice of Infection Control and Waste Management among Traditional Medicine Practitioners in Bhutan, 2019: A Nationwide Cross-Sectional Survey
Source: Evid Based Complement Alternat Med. 2021 Apr 12;2021:6691780. doi: 10.1155/2021/6691780 (PMC8055410; doi:10.1155/2021/6691780)
Supplement: Supplementary Materials — Study questionnaire of BTM infection control and waste management survey, 2019. [file 6691780.f1.pdf]

Data collection form (English)

Q	*Question*	*Responses*
	**BASIC DETAILS**	
G1	How old are you?	__________ years
G2	What is your sex?	☐Male<br>☐Female
G3	How many years have you been working?	__________ years
G4	What is your designation?	☐*Drungtsho*<br>☐*Smenpa*
G5	Which hospital do you work?	☐National Traditional Medicine Hospital<br>☐Regional Referral Hospital<br>☐Dzongkhag Hospital<br>☐BHU I<br>☐BHU II
G6	Do you have hand washing facilities at your hospital?	
	Running water	☐ Yes<br>☐ No
	Soap	☐ Yes<br>☐ No
	Sink / washing basin	☐ Yes<br>☐ No
G7	Was infection control taught during your training period?	☐ Yes<br>☐ No
G8	Did you get any training or workshop on infection control after joining service?	☐ Yes<br>☐ No
G9	Did you get vaccinated for hepatitis B?	☐ Yes<br>☐ No
	**KNOWLEDGE**	
K1	In the World Health Organisation hand washing guideline, how many steps are there in hand washing?	_____________ steps
K2	What are the five moments? of hand hygiene? (fill in the blanks)<br><br>*Answers:*<br>*before touching a patient,*<br>*before clean/aseptic procedures,*<br>*after body fluid exposure/risk,*<br>*after touching a patient, and.*<br>*after touching patient surroundings.*	1.<br>2.<br>3.<br>4.<br>5.
K3	If you are performing a blood-letting procedure, what type of gloves will you use in an ideal situation?	☐ Sterile/surgical gloves<br>☐ Clean gloves<br>☐ Utility gloves
K4	After performing a blood-letting procedure, where will you dispose your scalpel?	☐ Green bucket<br>☐ Red bucket<br>☐ Yellow box
K5	After performing a blood-letting procedure, where will you dispose the gauze material?	☐ Green bucket<br>☐ Red bucket

		☐ Blue bucket<br>☐ Yellow box		
K6	After performing a blood-letting procedure, what is the first step before you dispose the blood?	☐ Disinfect the blood<br>☐ Dispose it into the pit<br>☐ Wash it off with water<br>☐ Dispose it directly into the toilet pot		
K7	Which of the following steps in traditional medicine can spread microorganisms?	☐ Herbal bath<br>☐ Blood letting<br>☐ Dispensing medicines<br>☐ Cupping<br>☐ *Serkhap*		
K8	Which of the following are NOT personal protective equipment?	☐ Mask<br>☐ Patient register<br>☐ Medicines<br>☐ Gloves		
	**ATTITUDES**			
		Not interested	*Not sure*	*Very much interested*
A1	According to *smen-pai gyu-dru*, how interested are you in learning about infection control?			
A2	Are you interested to adopt the national guideline on infection control at your traditional medicine unit?			
A3	If there is a training on infection control, are you interested to participate in it?			
	**PRACTICES**			
		Never	*Sometimes*	*Always*
P1	How often do you wear apron at your workplace?			
P2	Before you read the pulse of a patient, how often do you wash your hands?			
P3	Before performing therapies, how often do you wash your hands?			
P4	How often do you wash hands after performing *num-tshug*?			
P5	How often do you dispose the scalpels in the correct dustbin?			
P6	Do you use a mask when you are suffering from common cold?			
P7	Do you use gloves when you dispense medicines?			
P8	Do you disinfect the instrument set used for cupping?			
P9	Do you examine patients in places other than your chamber?			
P10	Do you disinfect the floor of your traditional medicine unit?			

Data collection form (Dzongkha)

འབྲུག་ལུ་ ནང་པའི་དྲུང་འཚོ་དང་སྨན་པ་ཚུ་གི་ གཉན་ནད་བཀག་འཛིན་གྱི་ཡོན་ཏན་ བསམ་བློ་ ལག་ལེན་བརྟག་ཞིབ།

འདི་ རང་གིས་བཀང་གི་ནི་དྲི་བ་ཨིན། དགོས་མཁོ་དང་བསྟུན་ཏེ་ ལན་ཚུ་ རྟགས་དང་ ཡང་ན་ ས་སྟོང་བསུབ་གནང་།

Q	དྲི་བ།	གདམ་ཁ།
	གཞི་རྟེན་ཁ་གསལ།	
G1	ཁྱོད་ སྐྱེ་ལོ་ག་དེམ་ཅིག་སྨོ།	ལོ་_____ཨིན།ཨང་ཡིག་བཙུག་གནང་།))
G2	ཕོ་མོའི་དབྱེ་བ་ག་ཅི་སྨོ།	☐ཕོ།<br>☐མོ
G3	ཞི་གཡོག་ནང་ལུ་ ལོ་ག་དེ་ཅིག་ཡར་སོའི།	ལོ་_____ཡར་སོའི།
G4	སྨན་ཁང་ནང་ལུ་ ཞུགས་འགན་ག་ཅི་སྨོ།	☐དྲུང་འཚོ།<br>☐སྨན་པ།
G5	སྨན་ཁང་ དབྱེ་རིམ་ ག་ཅི་ནང་ཞུགས་ལཱ་གནངམ་སྨོ།	☐རྒྱལ་ཡོངས་ནང་པའི་སྨན་ཁང་།<br>☐ལུང་ཕྱོགས་གཙོ་བསྟེན་སྨན་ཁང་།<br>☐རྫོང་ཁག་སྨན་ཁང་།<br>☐གཞི་རྟེན་སྨན་ཁང་ སྡེ་རིམ་༡པ།<br>☐གཞི་རྟེན་སྨན་ཁང་ སྡེ་རིམ་༢པ།
G6	ཁྱོད་ཀྱི་སྨན་ཁང་ནང་ ལག་པ་འཁྱུ་ནི་གི་མཐུན་རྐྱེན་ཡོད་ག།	
	ཆུ།	☐ཡོད།<br>☐མེད།
	གླང་ལེབ།	☐ཡོད།<br>☐མེད།
	ཆུ་གཞོང་།	☐ཡོད།<br>☐མེད།
G7	སློབ་སྦྱོང་འབད་བའི་སྐབས་ གཉན་ནད་བཀག་འཛིན་ ལྷབ་ཅི་ག།	☐ཡོད།<br>☐མེད།
G8	ཞི་གཡོག་ནང་འཛུལ་ཞིནམ་ལས་ གཉན་ནད་བཀག་འཛིན་གྱི་ སྦྱོང་བརྡར་ཐོབ་ཅི་ག།	☐ཡོད།<br>☐མེད།

G9	མཆིན་ནད་ ཧེ་པ་ཊི་ཊིསི་བི་གི་ སྨན་ཁང་ཐོབ་ཅི་ག།	☐ཡོད།<br>☐མེད།
	ཡོན་ཏན།	
K1	འཛམ་གླིང་གསོ་བའི་ཚོགས་སྡེ་གི་ལམ་སྟོན་ནང་ལུ་ ལགཔ་འཁྱུ་ནི་ རིམ་པ་ ག་དེ་ཅིག་ཡོད།	རིམ་པ______ཡོད།
K2	སྨན་ཁང་ནང་ ལགཔ་ ནམ་ར་འཁྱུ་དགོཔ་སྨོ།	༡།<br>༢།<br>༣།<br>༤།<br>༥།
K3	གཏར་རྐྱབ་པའི་སྐབས་ ལག་ཤུབས་ག་ཅི་བཟུམ་ ལག་ལེན་འཐབ་སྨོ།	☐ཤག་བཅོས་ལག་ཤུབས།<br>☐ལག་ཤུབས་གཙང་ཏོག་ཏོ།<br>☐འབེད་སྤྱོད་ལག་ཤུབས།
K4	ཁྲག་ གཏར་བའི་ཤུལ་ལུ་ གཏར་གཞིའི་ཆ་བྱད་ ག་ཏེ་ལུ་བཀོཝ་སྨོ།	☐ཕྱག་སྙིགས་སྒྲོམ་ལྗང་ཁ།<br>☐ཕྱག་སྙིགས་སྒྲོམ་དམརཔོ།<br>☐ཕྱག་སྙིགས་སྒྲོམ་སེརཔོ།
K5	ཁྲག་གཏར་བའི་ཤུལ་ལུ་ རྨ་རས་ ག་ཏེ་བཀོཝ་སྨོ།	☐ཕྱག་སྙིགས་སྒྲོམ་ལྗང་ཁ།<br>☐ཕྱག་སྙིགས་སྒྲོམ་དམརཔོ།<br>☐ཕྱག་སྙིགས་སྒྲོམ་ཧོནམ།<br>☐ཕྱག་སྙིགས་སྒྲོམ་སེརཔོ།
K6	ཁྲག་ གཏར་བའི་ཤུལ་ལུ་ ཁྲག་དེ་ མ་བཀོ་བའི་ཧེ་མ་ ག་ཅི་ འབདཝ་སྨོ།	☐ཁྲག་ གཞན་སྲིན་སེལ་ནི།<br>☐ཕྱག་སྙིགས་བླུགས་སར་བཀོ་ནི།<br>☐ཆུ་ བཤལ་བཀོ་ནི།<br>☐ཆབ་གསང་ནང་བཀོ་ནི།
K7	གཉན་ནད་ཚུ་ འོག་ལུ་ཡོད་མི་ ག་ཅི་ལས་འབྱུཝ་སྨོ།	☐ཆུ་ལུམས་སྦྲངམ་ད།<br>☐ཁྲག་གཏརཝ་ད།<br>☐སྨན་སྦྱོདཔ་ད།<br>☐མི་འབུམ་རྐྱབ་ད།<br>☐གསེར་ཁབ་རྐྱབ་ད།

K8	འོག་ལུ་ཡོད་མི་རྩུ་ལས་ ག་དེ་ རང་སྒྲུང་ཅ་ཆས་ མེན་པས།	☐ཁ་རས།<br>☐ནད་པའི་བོ་དེབ།<br>☐སྨན།<br>☐ལག་ཤུབས།		
	བསམ་བློ།			
		སྤྲོ་བ་མེད།	ཐེ་ཚོམ་ནང་།	དང་འདོད་སྤྲོམ་ཡོད།
A1	ཁྱོད་ སྨན་པའི་རྒྱུ་དྲུག་དང་འབྲེལ་ གཉན་ནད་བཀག་འཛིན་ འབད་ནི་ དང་འདོད་ཡོད་ག།			
A2	ཁྱོད་ རྒྱལ་ཡོངས་གཉན་ནད་བཀག་འཛིན་ལམ་སྟོན་དེ་ བསྟར་ སྤྱོད་འབད་ནི་ དང་འདོད་ཡོད་ག།			
A3	གཉན་ནད་བཀག་འཛིན་གྱི་སྦྱོང་བརྡར་ཡོད་པ་ཅིན་ ཁྱོད་ བཅའ་ མར་གཏོགས་ནི་གི་ དང་འདོད་ཡོག་ད།			
	ལག་ལེན།			
		རྩ་ལས་མེད།	རེ་ཚེ་སྐབས།	རྟག་བུ་ར།
P1	ཁྱོད་ སྨན་ཁང་ནང་ལུ་ སྒྲིག་གོ་གྱོན་ནི་ཡོད་ག།			
P2	རྩ་མ་བལྟ་བའི་ཧེ་མ་ ལགཔ་འཁྱུ་ནི་ཡོད་ག།			
P3	དཔྱད་ཚུ་མ་འབད་བའི་ཧེ་མ་ ལགཔ་འཁྱུ་ནི་ཡོད་ག།			
P4	སྨུམ་ཚུགས་རྒྱབ་ཚར་བའི་ཤུལ་ལུ་ ལགཔ་འཁྱུ་ནི་ཡོད་ད།			
P5	གཏར་རྒྱབ་པའི་ཤུལ་ལུ་ གཏར་གའི་ཆ་བྱད་ ཕྱག་སྙིགས་སྒྲོམ་ ནང་ བཀོ་ནི་ཡོད་ག།			
P6	ཁྱོད་ ཚ་རིམས་ཐོབ་ད་ ཁ་རས་ལག་ལེན་འཐབ་ནི་ཡོད་ག།			
P7	ཁྱོད་ སྨན་སྦྱོདཔ་ད་ ལག་ཤུབས་བཙུག་ནི་ཡོད་ག།			
P8	མེ་འབུམ་རྒྱབ་ཚར་བའི་ཤུལ་ལུ་ ཆ་བྱད་ཚུ་ གཉན་སྲིན་སེལ་ནི་ ཡོད་ག།			
P9	ནདཔ་ཚུ་ སྨན་ཁང་གི་ཁང་མིག་གི་ཕྱི་ཁར་བལྟ་ནི་ཡོད་ག།			
P10	ཁྱོད་ཀྱིས་ སྨན་ཁང་དེ་ ཆུ་སྨན་གླུག་སྟེ་འཁྱུ་ནི་ཡོད་ག།			
